# Causal Roles of Sleep Duration in Osteoporosis and Cardiometabolic Diseases: A Mendelian Randomization Study

**DOI:** 10.1155/2022/6819644

**Published:** 2022-10-13

**Authors:** Bin He, Xiaojun Chen, Hao Liu, Muzi Zhang, Baoshan Yin, Lifeng Yin, Zhengxue Quan, Yunsheng Ou, Mingqi Sun, Yong Zhu, Hongwang Cui

**Affiliations:** ^1^Department of Orthopedics, The First Affiliated Hospital of Chongqing Medical University, Chongqing, China; ^2^Department of Orthopedics, The Affiliated Traditional Chinese Medicine Hospital of Southwest Medical University, Luzhou, Sichuan, China; ^3^Department of Orthopaedic Trauma, The Second Affiliated Hospital of Inner Mongolia Medical University, Huhhot, Inner Mongolia, China; ^4^Department of Spine and Osteopathic Surgery, The First Affiliated Hospital of Hainan Medical University, Haikou, China

## Abstract

Sleep duration suggests some association with osteoporosis and cardiometabolic diseases, but it is unknown if these associations are causal or confounded. In this two-sample Mendelian randomization (MR) study, we included the largest genome-wide association studies (GWASs) associated with sleep duration and the outcome measures of osteoporosis and cardiometabolic diseases. Finally, 25 single nucleotide polymorphisms (SNPs) associated with short sleep duration and 7 SNPs associated with long sleep duration obtained the genome-wide significance (*P* < 5 × 10^−8^) and were used as instrumental variables. Genetic predisposition to short sleep duration was strongly associated with increased risk of coronary artery disease (beta-estimate: 0.199, 95% confidence interval CI: 0.081 to 0.317, standard error SE:0.060, *P* value = 0.001) and heart failure (beta-estimate: 0.145, 95% CI: 0.025 to 0.264, SE:0.061, *P* value = 0.017), which were both confirmed by the sensitivity analyses. Both short and long sleep duration may reduce the estimated bone mineral density (eBMD, beta-estimate: -0.086, 95% CI: -0.141 to -0.031, SE:0.028, *P* value = 0.002 for short sleep duration; beta-estimate: -0.080, 95% CI: -0.120 to -0.041, SE:0.020, *P* value < 0.0001 for long sleep duration). There was limited evidence of associations between sleep duration and fracture, type 2 diabetes, atrial fibrillation, fasting glucose, fasting insulin, or HbA1c. This study provides robust evidence that short sleep duration is causally associated with high risk of coronary artery disease and heart failure and suggests that short sleep duration should be avoided to prevent these two cardiovascular diseases. Short and long sleep duration show some MR association with reduced eBMD, which indicates that both short and long sleep duration may be prevented to reduce the incidence of osteoporosis.

## 1. Introduction

Disturbed sleep duration widely occur in a modern society and poor sleep quality may increase the risk of various diseases including diabetes mellitus, cardiovascular, and metabolic diseases [1–5]. The detriment of short sleep duration on osteoporosis and cardiovascular diseases may be attributed to increased activity in sympathetic nervous system, inflammation, and accelerated atherosclerosis [6–10]. Long sleep duration may be also a fatal lifestyle factor [11–13], but few studies reported the mechanisms of adverse effects caused by long sleep duration [14].

Previous studies and meta-analysis studies reported that both short and long sleep duration had some association with osteoporosis, atrial fibrillation, and heart failure, but their results are conflicting [9, 15–20] and it is elusive whether these associations are causal or confounded. Osteoporosis and cardiometabolic diseases are highly polygenic traits based on current genome-wide association studies (GWAS) [21–25].

The two-sample Mendelian randomization (MR) study has emerged as an effective and powerful approach to explore the causal factors of diseases by using the GWAS summary statistics [26–30]. In this study, we use single nucleotide polymorphisms (SNPs) strongly associated with short (<7 h per night) and long (≥9 h per night) sleep duration as the instrumental variables and aim to explore the causal effect of sleep duration on osteoporosis and cardiometabolic diseases.

## 2. Methods

### 2.1. Genetic Instrument for Sleep Duration

This MR study was conducted based on publicly available summary-level data from GWASs (Table 1). The largest available GWAS meta-analysis reported genetic variants associated with sleep duration of European descent [31]. SNPs with genome-wide significance (*P* < 5 × 10^−8^) were thought to have robust association with sleep duration, including 27 SNPs associated with short sleep duration (<7 h; *n* = 106,192 cases/305,742 controls), and 8 SNPs associated with long sleep duration (≥9 h; *n* = 34,184 cases/305,742 controls).

SNPs used as instrumental variables should be not in linkage disequilibrium (LD), and thus we excluded two SNPs (rs75539574 and rs142180737) for short sleep duration and one SNP (rs549961083) for long sleep duration due to high LD (*r*^2^ ≥ 0.001). Finally, 25 SNPs associated with short sleep duration and 7 SNPs associated with long sleep duration served as instrumental variables (Supplementary Table 1). If SNPs were unavailable in the outcome dataset, the proxy SNPs in LD (*r*^2^ > 0.9) were highly correlated with the original SNPs and used as the instrumental variables (Supplementary Table 2).

### 2.2. Outcome Data Sources

Table 1 demonstrated the outcome data sources. Bone mineral density (BMD) as estimated by heel quantitative ultrasound and fracture among 426,824 people were applied to assess the osteoporosis. Fracture cases were defined as the break in the continuity of bone at any site apart from the skull, face, hands, feet, and pathological fractures due to malignancy, atypical femoral fractures, and periprosthetic and healed fracture codes [32]. In terms of cardiometabolic diseases, the outcome measures included coronary artery disease among 547,261 individuals [33], heart failure among 977,323 people [34], and atrial fibrillation among 587,446 persons [35]. Fasting glucose, fasting insulin, and HbA1c were included to assess the glycaemic traits in the large-scale GWAS among 281,416 individuals [36]. Summary statistics for the SNPs related to sleep duration and corresponding statistics of outcomes were presented in Supplementary Table 2. rs10068371 was used as a proxy for rs4585442 among all outcomes, while rs11236879 was used as a proxy for rs10899257 among fasting glucose, fasting insulin, and HbA1c. No proxy SNP was found for rs1380703 among eBMD.

### 2.3. Statistical Analyses

Inverse variance weighted (IVW) meta-analysis of the Wald ratio, weighted median, and MR-Egger regression methods were used to evaluate MR association between sleep duration and outcomes. MR pleiotropy residual sum and outlier test (MR-PRESSO) was also used to assess the presence of pleiotropic SNPs and the effect estimates were recalculated after removing SNP outliers [37–39]. The ethical approval was presented in the original publications, and all methods were performed based on relevant guidelines and regulations. *P* < 0.05 suggested statistically significant difference. All statistical analyses were performed in R V.4.0.4 software by using the R packages of ‘MendelianRandomization' [40], ‘TwoSampleMR' [41], and ‘MR-PRESSO' [42].

## 3. Results

### 3.1. Osteoporosis

**Figure 1:**
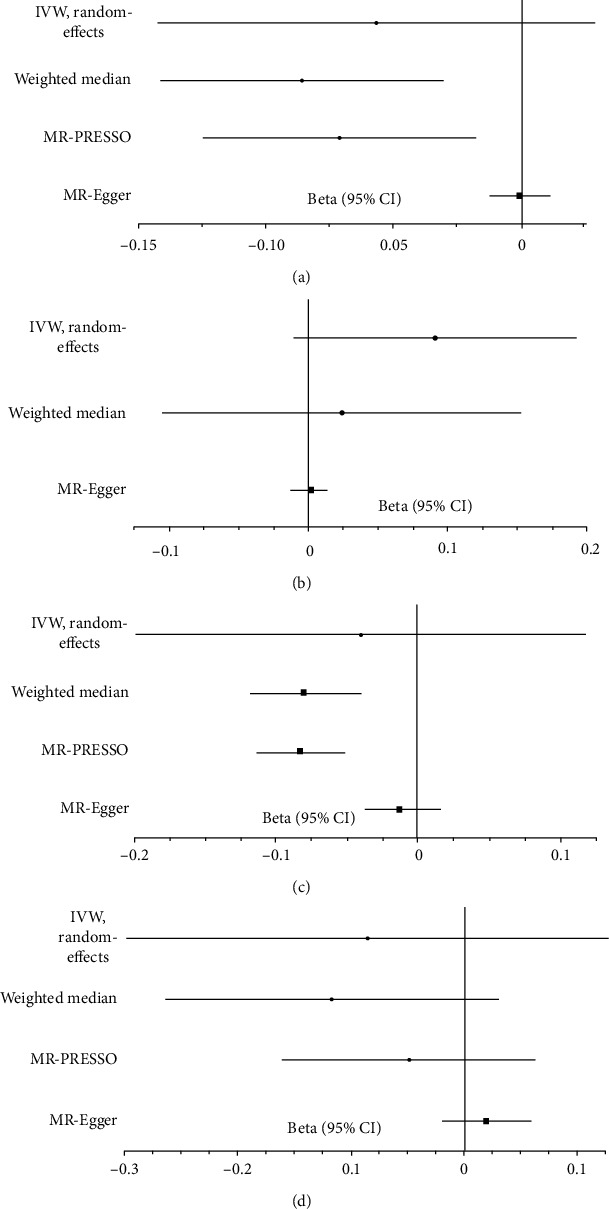
Beta (95% CIs) for association between sleep duration and osteoporosis. These effects represented the causal influence of short sleep duration on (a) eBMD and (b) fracture and long sleep duration on (c) eBMD and (d) fracture.

We evaluated the causal effect of sleep duration on eBMD and fracture in this MR analysis (Figure 1 and Table 2). According to weighted-median analysis, genetically short sleep duration were associated with low eBMD (beta-estimate: -0.086, 95% confidence interval CI: -0.141 to -0.031, standard error SE:0.028, *P* value = 0.002), but this significant finding was not supported in IVW analysis (beta-estimate: -0.057, 95% CI: -0.142 to 0.028, SE:0.044, *P* value = 0.191, Figure 1(a) and Table 2). Consistently, long sleep duration also showed significant MR association with low eBMD based on weighted-median analysis (beta-estimate: -0.080, 95% CI: -0.120 to -0.041, SE:0.020, *P* value < 0.0001), which was not confirmed by IVW analysis (beta-estimate: -0.040, 95% CI: -0.199 to 0.118, SE:0.081, *P* value = 0.618, Figure 1(c) and Table 3).

According to IVW analysis, sleep duration showed no significant MR association with fracture (beta-estimate: 0.091, 95% CI: -0.011 to 0.192, SE:0.052, *P* value = 0.081 for short sleep duration, Figure 1(b); beta-estimate: -0.086, 95% CI: -0.300 to 0.127, SE:0.109, *P* value = 0.429 for long sleep duration, Figure 1(d)). These results were also confirmed by weighted-median analyses (beta-estimate: 0.024, 95% CI: -0.104 to 0.153, SE:0.065, *P* value = 0.708 for short sleep duration, beta-estimate: -0.118, 95% CI: -0.266 to 0.030, SE:0.076, *P* value = 0.118 for long sleep duration, Table 2 and Table 3).

### 3.2. Cardiometabolic Diseases

**Figure 2:**
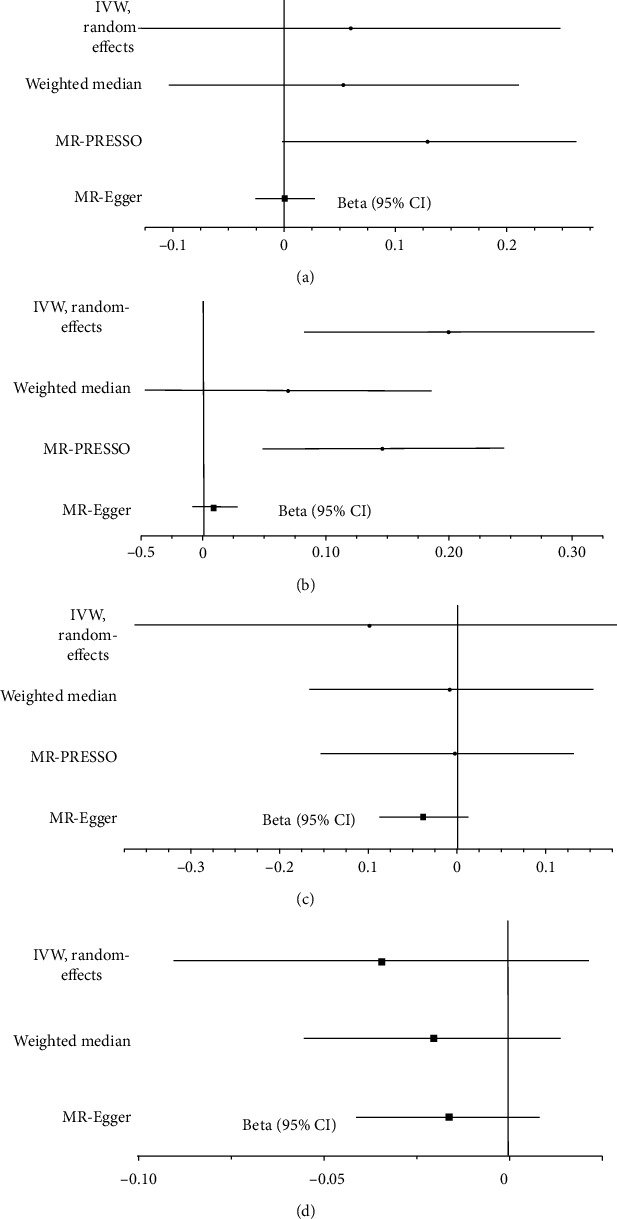
Beta (95% CIs) for association between sleep duration and type 2 diabetes/coronary artery disease. These effects represented the causal influence of short sleep duration on (a) type 2 diabetes and (b) coronary artery disease and long sleep duration on (c) type 2 diabetes and (d) coronary artery disease.

**Figure 3:**
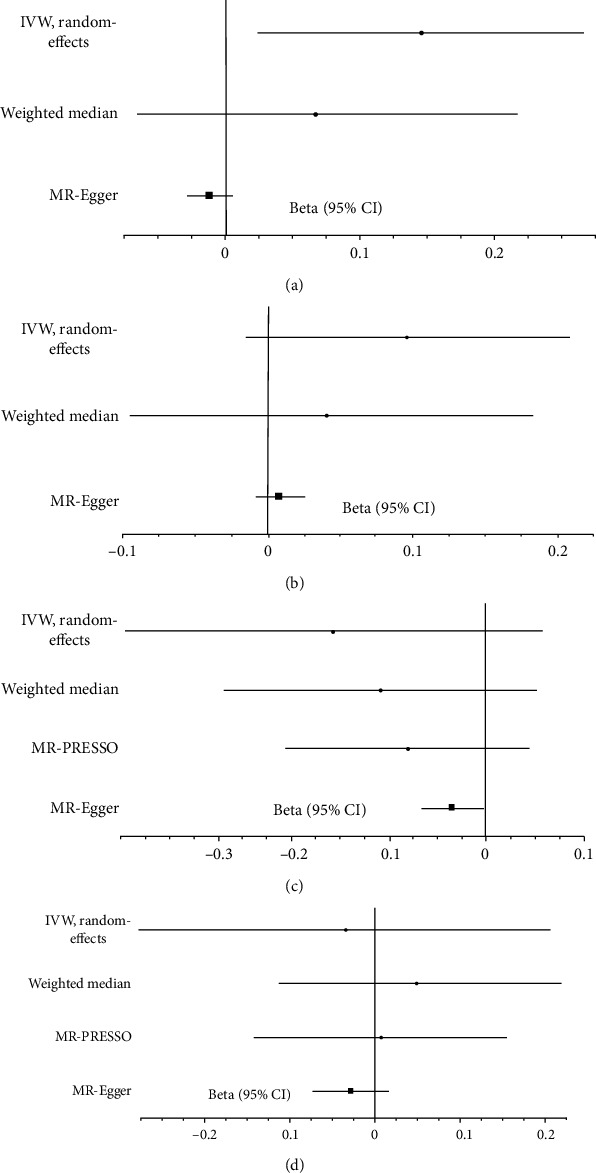
Beta (95% CIs) for association between sleep duration and type 2 diabetes/coronary artery disease. These effects represented the causal influence of short sleep duration on (a) heart failure and (b) atrial fibrillation and long sleep duration on (c) heart failure and (d) atrial fibrillation.

This MR analysis included outcome measures of type 2 diabetes, coronary artery disease, heart failure, and atrial fibrillation. The IVW analyses found the significant causal influence of short sleep duration on increased risk of coronary artery disease (beta-estimate: 0.199, 95% CI: 0.081 to 0.317, SE:0.060, *P* value = 0.001, Figure 2(b)) and heart failure (beta-estimate: 0.145, 95% CI: 0.025 to 0.264, SE:0.061, *P* value = 0.017, Figure 3(a) and Table 2). These positive results were not supported by weighted-median analyses. Short sleep duration revealed no causal effect on type 2 diabetes (beta-estimate: 0.06, 95% CI: -0.127 to 0.247, SE:0.095, *P* value = 0.528, Figure 2(a)) or atrial fibrillation (beta-estimate: 0.096, 95% CI: -0.015 to 0.207, SE:0.057, *P* value = 0.090, Figure 3(b) and Table 2), which were also confirmed by weighted-median analyses.

In addition, The IVW analyses showed that long sleep duration demonstrated no MR association with type 2 diabetes (beta-estimate: -0.100, 95% CI: -0.384 to 0.184, SE:0.145, *P* value = 0.488, Figure 2(c)), coronary artery disease (beta-estimate: -0.034, 95% CI: -0.090 to 0.022, SE:0.028, *P* value = 0.233, Figure 2(d)), heart failure (beta-estimate: -0.157, 95% CI: -0.371 to -0.057, SE:0.109, *P* value = 0.152, Figure 3(c)) or atrial fibrillation (beta-estimate: -0.034, 95% CI: -0.277 to 0.209, SE:0.124, *P* value = 0.785, Figure 3(d)). These results were all confirmed by weighted-median analyses (Table 3).

### 3.3. Glycaemic Traits

**Figure 4:**
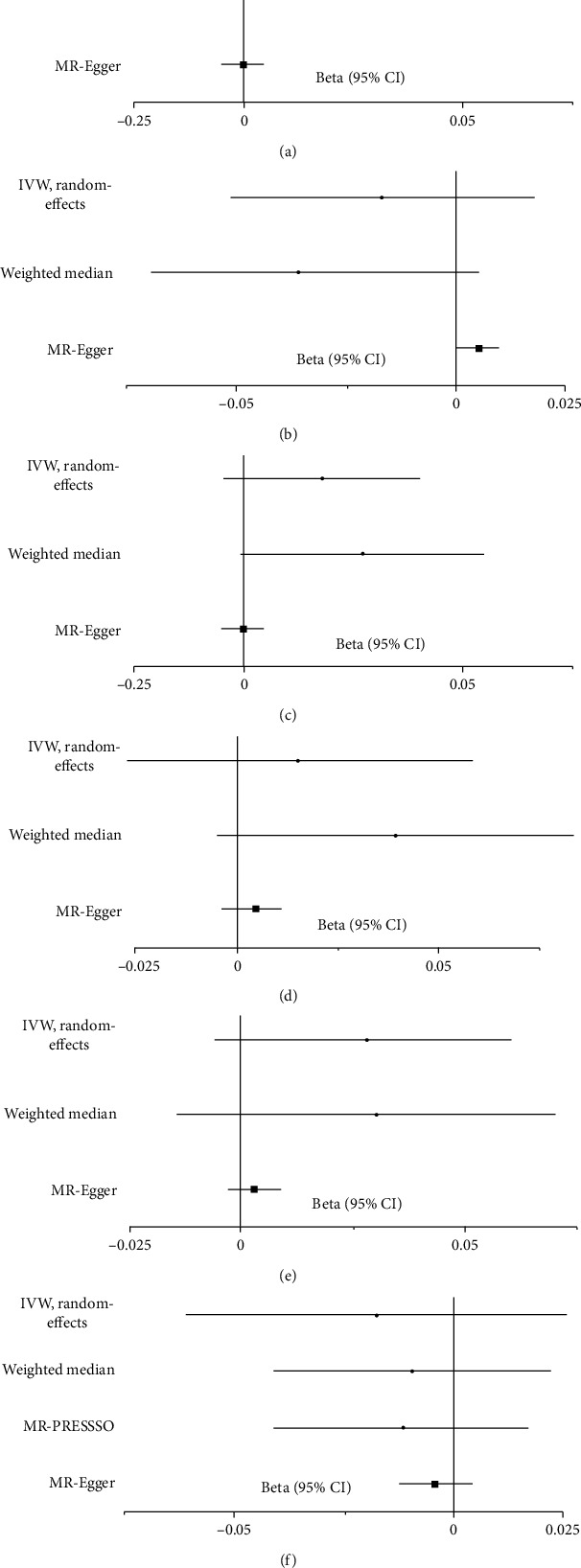
Beta (95% CIs) for association between sleep duration and type 2 diabetes/coronary artery disease. These effects represented the causal influence of short sleep duration on (a) fasting glucose, (b) fasting insulin, and (c) HbA1c and long sleep duration on (d) fasting glucose, (e) fasting insulin, and (f) HbA1c.

Fasting glucose, fasting insulin, and HbA1c were involved to evaluate the glycaemic traits after the intervention of sleep duration (Table 2, Table 3, and Figure 4). Based on the results of IVW analysis, short sleep duration showed no obvious MR association with fasting glucose (beta-estimate: 0.022, 95% CI: -0.012 to 0.055, SE:0.017, *P* value = 0.205, Figure 4(a)), fasting insulin (beta-estimate: -0.017, 95% CI: -0.051 to 0.017, SE:0.017, *P* value = 0.320, Figure 4(b)), or HbA1c (beta-estimate: 0.017, 95% CI: -0.005 to 0.039, SE:0.011, *P* value = 0.127, Figure 4(c) and Table 2). Long sleep duration also demonstrated no causal effect on fasting glucose (beta-estimate: 0.015, 95% CI: -0.027 to 0.058, SE:0.022, *P* value = 0.484, Figure 4(d)), fasting insulin (beta-estimate: 0.028, 95% CI: -0.005 to 0.060, SE:0.017, *P* value = 0.094, Figure 4(e)), or HbA1c (beta-estimate: -0.017, 95% CI: -0.060 to 0.026, SE:0.022, *P* value = 0.443, Figure 4(f) and Table 3). These findings were also confirmed by the weighted-median analyses (Table 2 and Table 3).

### 3.4. Evaluation of Assumptions and Sensitivity Analyses

Little evidence of directional pleiotropy was found for all models only except for the analyses between short sleep duration and fasting insulin (MR-Egger intercept *P* value = 0.048) and between long sleep duration and heart failure (MR-Egger intercept *P* value = 0.029) (Table 2). Among the 25 SNP instrumental variables associated with short sleep duration, MR-PRESSO method only identified 11 outliers (rs12567114, rs2863957, rs2014830, rs13107325, rs4585442, rs9367621, rs1229762, rs7939345, rs59779556, rs12963463, and rs5757675) for eBMD, one outlier (rs3776864) for type 2 diabetes, and two outliers (rs2820313 and rs11763750) for coronary artery disease. For the 7 SNP instrumental variables associated with long sleep duration, MR-PRESSO method identified two outliers (rs17817288 and rs17688916) for eBMD, two outliers (rs17817288 and rs17688916) for fracture, one outlier (rs17817288) for type 2 diabetes, one outlier (rs17817288) for heart failure, two outliers (rs75458655 and rs17688916) for atrial fibrillation, and two outliers (rs17817288, rs17688916) for HbA1c (Table 4).

After excluding these outlying SNP variants, these remarkable MR associations were confirmed between short sleep duration and decreased eBMD (beta-estimate: -0.071, 95% CI: -0.125 to -0.018, SE:0.027, *P* value = 0.008, Figure 1(a)), between short sleep duration and increased risk of coronary artery disease (beta-estimate: 0.145, 95% CI: 0.048 to 0.243, SE:0.050, *P* value = 0.003, Figure 2(b)Table 4). In addition, long sleep duration was also confirmed to reduce the eBMD (beta-estimate: -0.083, 95% CI: -0.114 to -0.051, SE:0.016, *P* value < 0.0001, Figure 1(c)), and the MR association of other outcomes were not changed after excluding the outlying SNP variants (Table 4).

## 4. Discussion

Our MR study found the robust causal roles of short sleep duration in increased risk of coronary artery disease and heart failure, which were confirmed by the sensitivity analyses. These suggested that short sleep duration should be avoided to prevent these two cardiovascular diseases, and long sleep duration did not reveal a protective role in them. In addition, both short and long sleep duration showed some MR association with decreased eBMD, suggesting that short and long sleep duration should be prevented to reduce the incidence of osteoporosis. We found no causal effect of sleep duration on fracture, type 2 diabetes, atrial fibrillation, fasting glucose, fasting insulin, or HbA1c. Our findings indicated that both short and long sleep duration should be avoided to decrease the incidence of cardiovascular diseases and osteoporosis.

Osteoporosis, a progressive systemic skeletal disease, is featured by low bone mass, microstructure deterioration of bone tissue, reduced BMD and bone strength which may result in the increased risk of fracture [43–45]. One meta-analysis included five cross-sectional studies and one prospective cohort study with 31,625 individuals, and it revealed that only long sleep duration may increase the risk of osteoporosis in middle-aged and elderly patients, and these findings were not consistent in short sleep duration [16]. Another observational study recruited 602 women aged 18-80 years and found an association between short sleep duration and low BMD [46]. Considering these inconsistent results, MR studies have been developed to ascertain causes of disease since they utilize genetic variants of exposure randomly assigned at conception, hence are less vulnerable to confounding factors than observational studies [47, 48].

To our knowledge, this is the first two-sample MR study to explore the causal relationship between sleep duration and eBMD. Our results found that both short and long sleep duration were remarkably associated with low eBMD, which were confirmed by the sensitivity analysis. Several mechanisms may contribute to low BMD caused by short sleep duration. Sleep deprivation can lead to disturbed endocrine and metabolic function, reduced secretion of growth hormone, and increased release of cortisol and estrogen levels [49, 50]. Proinflammatory responses are overactivated by short sleep duration and obviously affects bone resorption [46]. In addition, long sleep duration shows some detrimental influence on insulin resistance and insulin sensitivity [51–53]. Reduced physical activity and mechanical loading caused by long sleep duration is also a significant risk for osteoporosis [54, 55].

Conventional observational studies reported conflicting results between sleep duration and the risk of many cardiovascular diseases [56–58]. One recent MR study included the summary GWAS of 335,410 individuals for sleep duration and a 30,482 population for coronary heart disease, and the results found no causal association between sleep duration and coronary heart disease [59]. Another MR study selected 7 SNPs associated with sleep duration as the instrumental variables and used the GWAS summary data of 185,305 people for coronary heart disease and 171,873 individuals for myocardial infarction. No causal associations were seen between sleep duration and these two vascular diseases [60]. However, the MR association between short sleep duration and increased risk of myocardial infarction was confirmed by one MR analysis [61].

In this two-sample study, we used 27 SNPs associated with short sleep duration and 8 SNPs associated with long sleep duration as the instrumental variables, and more larger patient samples were included for the summary GWAS data of cardiovascular outcomes (Table 1). Our results revealed the important causal effect of short sleep duration on increased risk of coronary heart disease and heart failure, which were also confirmed by the sensitivity analysis. Long sleep duration did not provide the protective role for the incidence of coronary heart disease and heart failure. Several mechanisms may account for the harmful effect of short sleep duration on coronary heart disease and heart failure, and they mainly included dysfunction of sympathetic nervous system, acceleration of metabolic diseases and atherosclerosis and cardiac dysfunction [7, 8, 62].

In addition, our results demonstrated that genetically predicted long sleep duration was unlikely to be causally associated with coronary heart disease, heart failure, atrial fibrillation, type 2 diabetes, fasting glucose, fasting insulin, or HbA1c. The association between long sleep duration and cardiovascular diseases in observational studies may be subjected to reverse causality and residual confounding [63], which can be effectively avoided in this MR study. In addition, there was substantial overlap in genetic influence on the association between negative life events (e.g. financial or relationship problems) and poor sleep quality, indicating the importance of gene–environment correlation to affect sleep-related diseases including cardiovascular diseases, osteoporosis, metabolic syndrome, and mortality [5, 9, 11, 61].

We also should consider some limitations. Firstly, all the included participants are of European origin, and it is unknown that whether our findings are applicable to other populations. Secondly, the contribution of short sleep duration to low eBMD was not translated to increased incidence of fracture, which requires more studies to explore their mechanisms. Thirdly, there may be some overlap patient sample between sleep duration and outcomes, which may have some influence on the results.

## 5. Conclusion

This two-sample MR study provides strong evidence to confirm that short sleep duration is a significantly causal risk factor of coronary heart disease and heart failure, and it also reveals some causal effect of short and long sleep duration on low eBMD, which may provide new insights to prevent cardiovascular diseases and osteoporosis.

## Figures and Tables

**Table 1:** Details of studies and datasets used for analyses.

	Traits	Samples size	Population	Consortium or cohort study (link URL)
Exposure	Short sleep duration	411,934	European	UK Biobank
	Long sleep duration	339,926	European	

Osteoporosis	eBMD	426,824	European	GEFOS (http://www.gefos.org)
	Fracture	426,824	European	

Cardiometabolic diseases	Type 2 diabetes	898,130	European	DIAGRAM (http://diagram-consortium.org)
	Coronary artery disease	547,261	European	UK Biobank and CARDIoGRAMplusC4D (https://cvd.hugeamp.org/)
	Heart failure	977,323	European	UK Biobank (http://www.broadcvdi.org/)
	Atrial fibrillation	587,446	Mixed	Meta-analysis of more than 50 studies (http://www.broadcvdi.org/)

Glycaemic traits	Fasting glucose	281,416	Mixed	MAGIC (https://magicinvestigators.org)
	Fasting insulin	281,416	Mixed	
	HbA1c	281,416	Mixed	

eBMD estimated bone mineral density.

**Table 2:** Mendelian randomization estimates of short sleep duration on outcomes.

Variables	IVW							Weighted median				MR-egger							
	Estimate	SE	95% CI	*P* value	*Q* value	*I* ^2^	Heterogeneity *P* value	Estimate	SE	95% CI	*P* value	Estimate	SE	95% CI	*P* value	Intercept	SE	95% CI	Pleiotropy *P* value
Osteoporosis																			
eBMD	-0.057	0.044	-0.142,0.028	0.191	273.599	91.60%	<0.001	-0.086	0.028	-0.141,-0.031	0.002	-0.026	0.175	-0.370,0.317	0.881	-0.001	0.006	-0.013,0.011	0.856
Fracture	0.091	0.052	-0.011,0.192	0.081	33.645	28.70%	0.091	0.024	0.065	-0.104,0.153	0.708	0.059	0.213	-0.359,0.477	0.782	0.001	0.007	-0.013,0.015	0.878
Cardiometabolic disease																			
Type 2 diabetes	0.06	0.095	-0.127,0.247	0.528	87.506	72.60%	<0.001	0.054	0.079	-0.102,0.209	0.499	0.032	0.399	-0.749,0.813	0.936	0.001	0.013	-0.025,0.027	0.942
Coronary artery disease	0.199	0.060	0.081,0.317	0.001	60.952	60.60%	<0.001	0.068	0.059	-0.048,0.184	0.249	-0.037	0.245	-0.518,0.444	0.880	0.008	0.008	-0.008,0.024	0.321
Heart failure	0.145	0.061	0.025,0.264	0.017	31.764	24.40%	0.133	0.067	0.077	-0.083,0.218	0.381	0.479	0.250	-0.010,0.968	0.055	-0.012	0.008	-0.028,0.005	0.168
Atrial fibrillation	0.096	0.057	-0.015,0.207	0.090	32.669	26.50%	0.111	0.041	0.072	-0.100,0.182	0.568	-0.109	0.235	-0.569,0.352	0.643	0.007	0.008	-0.008,0.023	0.369
Glycaemic traits																			
Fasting glucose	0.022	0.017	-0.012,0.055	0.205	47.006	48.90%	0.003	0.029	0.019	-0.008,0.066	0.120	0.016	0.073	-0.127,0.159	0.825	0.000	0.002	-0.005,0.005	0.937
Fasting insulin	-0.017	0.017	-0.051,0.017	0.320	37.924	36.70%	0.035	-0.036	0.021	-0.077,0.005	0.085	-0.150	0.069	-0.285,-0.015	0.030	0.005	0.002	0.000,0.009	0.048
HbA1c	0.017	0.011	-0.005,0.039	0.127	36.964	35.10%	0.044	0.026	0.014	-0.001,0.053	0.063	0.039	0.047	-0.053,0.131	0.410	-0.001	0.002	-0.004,0.002	0.636

eBMD: estimated bone mineral density, SE: standard error, CI: confidence interval.

**Table 3:** Mendelian randomization estimates of long sleep duration on outcomes.

Variables	IVW							Weighted median				MR-Egger							
	Estimate	SE	95% CI	*P* value	*Q* value	*I* ^2^	Heterogeneity Pvalue	Estimate	SE	95% CI	*P* value	Estimate	SE	95% CI	*P* value	Intercept	SE	95% CI	Pleiotropy *P* value
Osteoporosis																			
eBMD	-0.04	0.081	-0.199,0.118	0.618	206.379	97.10%	<0.001	-0.080	0.020	-0.120,-0.041	<0.001	0.143	0.233	-0.314,0.600	0.540	-0.013	0.016	-0.043,0.017	0.400
Fracture	-0.086	0.109	-0.300,0.127	0.429	29.658	79.80%	<0.001	-0.118	0.076	-0.266,0.030	0.118	-0.358	0.309	-0.964,0.248	0.246	0.019	0.021	-0.021,0.060	0.346
Cardiometabolic disease																			
Type 2 diabetes	-0.1	0.145	-0.384,0.184	0.488	38.170	84.30%	<0.001	-0.008	0.081	-0.166,0.150	0.921	0.409	0.385	-0.346,1.163	0.288	-0.036	0.025	-0.085,0.014	0.158
Coronary artery disease	-0.034	0.028	-0.090,0.022	0.233	18.655	67.80%	0.005	-0.020	0.018	-0.055,0.014	0.244	0.183	0.170	-0.150,0.516	0.281	-0.016	0.013	-0.041,0.008	0.196
Heart failure	-0.157	0.109	-0.371,0.057	0.152	19.285	68.90%	0.004	-0.108	0.081	-0.268,0.051	0.184	0.354	0.249	-0.134,0.841	0.155	-0.036	0.016	-0.067,-0.004	0.029
Atrial fibrillation	-0.034	0.124	-0.277,0.209	0.785	29.737	79.80%	<0.001	0.049	0.083	-0.113,0.212	0.552	0.372	0.356	-0.325,1.070	0.295	-0.028	0.023	-0.073,0.017	0.225
Glycaemic traits																			
Fasting glucose	0.015	0.022	-0.027,0.058	0.484	13.993	57.10%	0.030	0.039	0.023	-0.005,0.083	0.083	-0.045	0.063	-0.169,0.078	0.475	0.004	0.004	-0.004,0.012	0.309
Fasting insulin	0.028	0.017	-0.005,0.060	0.094	2.438	0.00%	0.875	0.030	0.021	-0.011,0.070	0.148	-0.016	0.048	-0.110,0.078	0.739	0.003	0.003	-0.003,0.009	0.330
HbA1c	-0.017	0.022	-0.060,0.026	0.443	25.457	76.40%	<0.001	-0.009	0.016	-0.039,0.022	0.581	0.036	0.066	-0.094,0.166	0.589	-0.004	0.004	-0.012,0.005	0.399

eBMD: estimated bone mineral density, SE: standard error, CI: confidence interval.

**Table 4:** Mendelian randomization estimates between sleep duration and outcomes after excluding outliers detected by MR-PRESSO.

	Estimate	SE	95% CI	*P* value
Short sleep duration				
eBMD excluding 11 outliers (rs12567114, rs2863957, rs2014830, rs13107325, rs4585442, rs9367621, rs1229762, rs7939345, rs59779556, rs12963463, rs5757675)	-0.071	0.027	-0.125,-0.018	0.008
Type 2 diabetes excluding one outlier (rs3776864)	0.129	0.066	-0.001,0.260	0.051
Coronary artery disease excluding two outliers (rs2820313, rs11763750)	0.145	0.050	0.048,0.243	0.003
Long sleep duration				
eBMD excluding two outliers (rs17817288, rs17688916)	-0.083	0.016	-0.114,-0.051	<0.001
Fracture excluding two outliers (rs17817288, rs17688916)	-0.05	0.057	-0.162,0.061	0.377
Type 2 diabetes excluding one outlier (rs17817288)	-0.002	0.078	-0.155,0.150	0.977
Heart failure excluding one outlier (rs17817288)	-0.081	0.064	-0.207,0.044	0.204
Atrial fibrillation excluding two outliers (rs75458655, rs17688916)	0.007	0.076	-0.142,0.156	0.923
HbA1c excluding two outliers (rs17817288, rs17688916)	-0.011	0.015	-0.040,0.017	0.444

eBMD: estimated bone mineral density, SE: standard error, CI: confidence interval.

## Data Availability

Data supporting the findings of this study were available within the paper.

## Supplementary Materials

Supplementary 1 Supplementary Table 1. Summary statistics of the single-nucleotide polymorphisms associated with sleep duration.

Supplementary 2 Supplementary Table 2. Summary statistics of the single-nucleotide polymorphisms associated with sleep duration and outcomes.
