# Supplementary material for: Causal Roles of Sleep Duration in Osteoporosis and Cardiometabolic Diseases: A Mendelian Randomization Study
Source: Biomed Res Int. 2022 Oct 13;2022:6819644. doi: 10.1155/2022/6819644 (PMC9586149; doi:10.1155/2022/6819644)
Supplement: Supplementary 1 — Supplementary Table 1. Summary statistics of the single-nucleotide polymorphisms associated with sleep duration. [file 6819644.f1.pdf]

Supplementary Table 1. Summary statistics of the single-nucleotide polymorphisms associated with sleep duration

Trait	SNP	Chr	Position	Effect alllele	Other allele	Beta	SE	P value
Short sleep	rs12567114	1	98527951	G	A	0.036	0.007	4.10E-09
Short sleep	rs2186122	1	66470206	T	A	0.024	0.006	4.80E-09
Short sleep	rs2820313	1	201870221	G	A	0.031	0.006	2.30E-09
Short sleep	rs7524118	1	34736052	C	T	0.03	0.006	4.90E-08
Short sleep	rs1380703	2	57941287	G	A	0.035	0.006	1.60E-11
Short sleep	rs2863957	2	114089551	C	A	0.054	0.007	2.60E-18
Short sleep	rs75539574	2	58871658	A	C	0.045	0.011	8.40E-11
Short sleep	rs2014830	3	50172397	C	T	0.03	0.006	2.70E-08
Short sleep	rs13107325	4	103188709	T	C	0.075	0.011	2.50E-13
Short sleep	rs17005118	4	82288564	A	G	0.03	0.007	2.50E-09
Short sleep	rs12518468	5	7249696	C	T	0.031	0.006	8.50E-09
Short sleep	rs3776864	5	102327868	A	C	0.031	0.006	1.70E-08
Short sleep	rs4585442	5	13550838	G	A	0.031	0.006	8.10E-10
Short sleep	rs12661667	6	41792545	T	C	0.028	0.007	2.80E-08
Short sleep	rs142180737	6	28344731	C	T	0.154	0.032	4.40E-09
Short sleep	rs9321171	6	129848635	C	T	0.031	0.006	4.20E-08
Short sleep	rs9367621	6	55040290	T	A	0.024	0.006	1.60E-08
Short sleep	rs11763750	7	2080114	G	A	0.035	0.008	5.10E-09
Short sleep	rs1229762	7	114218582	T	C	0.037	0.003	1.10E-12
Short sleep	rs60882754	8	52886619	A	T	0.055	0.012	1.80E-08
Short sleep	rs1607227	11	28808617	G	T	0.031	0.007	1.50E-09
Short sleep	rs7939345	11	47980568	T	G	0.035	0.007	4.00E-08
Short sleep	rs17388803	15	48027204	C	A	0.053	0.01	6.50E-10
Short sleep	rs59779556	16	56227965	T	G	0.025	0.006	2.00E-08
Short sleep	rs205024	17	11227352	C	T	0.031	0.006	2.70E-08
Short sleep	rs12963463	18	53099093	C	T	0.029	0.006	1.9E-11
Short sleep	rs5757675	22	39838892	G	T	0.034	0.007	2.7E-09
Long sleep	rs7534398	1	7767464	A	T	0.047	0.012	2.10E-08
Long sleep	rs6737318	2	114083120	G	A	0.076	0.011	3.40E-13
Long sleep	rs549961083	5	58184093	T	C	0.534	0.117	9.60E-09
Long sleep	rs10899257	11	76415209	A	G	0.068	0.013	4.60E-08
Long sleep	rs3751046	11	122828342	G	A	0.07	0.013	2.00E-08
Long sleep	rs75458655	11	118115331	T	C	0.185	0.029	5.40E-12
Long sleep	rs17817288	16	53807764	A	G	0.039	0.009	8.90E-09
Long sleep	rs17688916	17	43778680	T	A	0.071	0.012	1.10E-11
