# Supplementary material for: Causal Roles of Sleep Duration in Osteoporosis and Cardiometabolic Diseases: A Mendelian Randomization Study
Source: Biomed Res Int. 2022 Oct 13;2022:6819644. doi: 10.1155/2022/6819644 (PMC9586149; doi:10.1155/2022/6819644)
Supplement: Supplementary 2 — Supplementary Table 2. Summary statistics of the single-nucleotide polymorphisms associated with sleep duration and outcomes. [file 6819644.f2.pdf]

Supplementary Table 2. Summary statistics of the single-nucleotide polymorphisms associated with sleep duration and outcomes

		Sleep duration			eBMD			Fracture			Type 2 diabetes			Coronary artery			Heart failure			Atrial fibrillation			Fasting glucose			Fasting insulin			HbA1C		
Sleep duration	SNP	Beta	SE	P value	Beta	SE	P value	Beta	SE	P value	Beta	SE	P value	Beta	SE	P value	Beta	SE	P value	Beta	SE	P value	Beta	SE	P value	Beta	SE	P value	Beta	SE	P value
Short sleep	rs12567114	0.036	0.007	4.10E-09	-0.0091	0.00206	5.10E-05	0.00144	0.00735	0.86	0.0049	0.0084	5.60E-01	-0.0008	0.0064	8.94E-01	-0.0069	0.0088	4.36E-01	-0.0005	0.008	9.48E-01	-0.0042	0.0021	1.10E-01	-0.0047	0.0025	8.82E-02	-0.0026	0.0016	9.27E-02
Short sleep	rs2186122	0.024	0.006	4.80E-09	0.00206	0.00186	5.20E-01	3.8E-05	0.00663	0.95	-0.0011	0.0075	8.80E-01	0.0132	0.0056	1.96E-02	0.0014	0.0079	8.61E-01	0.0142	0.0071	4.59E-02	-0.0029	0.0018	1.25E-01	-0.0019	0.002	4.54E-01	-0.0011	0.0013	3.35E-01
Short sleep	rs2820313	0.031	0.006	2.30E-09	-0.0016	0.00193	2.60E-01	0.0035	0.00689	0.68	0.011	0.0079	1.50E-01	0.0294	0.006	8.85E-07	0.0122	0.0083	1.42E-01	0.0157	0.0077	4.04E-02	2.00E-04	0.0019	9.59E-01	-0.0019	0.0022	2.03E-01	0.002	0.0014	1.75E-01
Short sleep	rs7524118	0.03	0.006	4.90E-08	-0.0005	0.00201	7.40E-01	-0.0058	0.00719	0.48	0.0069	0.0081	3.90E-01	6.00E-04	0.0061	9.26E-01	-0.0014	0.0085	8.66E-01	-0.0064	0.0078	4.16E-01	0.0022	0.002	4.95E-01	-0.001	0.0022	1.90E-01	0.0017	0.0015	4.06E-01
Short sleep	rs1380703	0.035	0.006	1.60E-11	_	_	_	0.00078	0.00686	0.9	-0.0098	0.0078	2.10E-01	-5.00E-04	0.006	9.38E-01	-0.0016	0.0083	8.46E-01	0.0019	0.0077	8.02E-01	0.0013	0.002	2.37E-01	-0.0006	0.0023	9.50E-01	0.0028	0.0015	8.29E-02
Short sleep	rs2863957	0.054	0.007	2.60E-18	0.00723	0.00221	3.20E-03	-0.0079	0.00791	0.23	-0.0085	0.009	3.40E-01	0.002	0.0068	7.69E-01	0.0112	0.0095	2.41E-01	-0.0022	0.0087	7.96E-01	0.0034	0.0022	6.14E-01	-0.0027	0.0025	2.23E-01	0.0032	0.0016	9.84E-02
Short sleep	rs2014830	0.03	0.006	2.70E-08	0.01534	0.00204	5.60E-13	-0.0008	0.00723	0.9	0.024	0.0081	2.70E-03	0.0116	0.0056	3.98E-02	0.0087	0.0085	3.06E-01	0.012	0.0078	1.24E-01	-0.0043	0.0018	4.86E-02	0.0019	0.002	3.14E-01	0.0029	0.0014	7.70E-02
Short sleep	rs13107325	0.075	0.011	2.50E-13	-0.0162	0.00352	2.50E-05	0.02669	0.01247	0.033	0.0018	0.015	9.00E-01	-3.00E-04	0.0112	9.76E-01	0.0701	0.017	3.97E-05	3.00E-04	0.0149	9.86E-01	-0.0031	0.0038	3.05E-01	-0.0102	0.0044	9.37E-03	-0.0021	0.0028	6.86E-01
Short sleep	rs17005118	0.03	0.007	2.50E-09	-0.0021	0.00209	5.80E-01	0.01041	0.00742	0.15	0.0084	0.0085	3.20E-01	0.0176	0.0057	2.14E-03	0.0174	0.0088	4.78E-02	0.0154	0.008	5.64E-02	0.0015	0.0019	6.78E-01	0.0052	0.0021	8.58E-03	0.0018	0.0015	5.60E-01
Short sleep	rs12518468	0.031	0.006	8.50E-09	-0.0059	0.00197	3.50E-03	0.00222	0.00697	0.71	-0.0071	0.0079	3.70E-01	-0.002	0.0061	7.39E-01	6.00E-04	0.0084	9.45E-01	-0.0035	0.0076	6.48E-01	0.0053	0.002	2.82E-02	0.0014	0.0022	5.50E-01	-0.0011	0.0015	4.17E-01
Short sleep	rs3776864	0.031	0.006	1.70E-08	-0.0058	0.00196	1.96E-03	0.01229	0.00695	0.074	-0.052	0.0079	4.30E-11	0.0076	0.0062	2.24E-01	0.0021	0.0085	8.02E-01	-0.0132	0.0077	8.79E-02	0.0062	0.002	9.82E-04	6.00E-04	0.0023	5.58E-01	-0.0017	0.0015	1.83E-01
Short sleep	*rs4585442	0.031	0.006	8.10E-10	-0.0118	0.002	1.10E-06	0.02405	0.0071	0.00068	0.0096	0.0081	2.40E-01	0.0034	0.0061	0.581	0.002	0.0085	0.8126	-0.0118	0.0077	1.26E-01	9.00E-04	0.002	9.60E-01	0.0036	0.0022	5.19E-02	7.00E-04	0.0015	7.53E-01
Short sleep	rs12661667	0.028	0.007	2.80E-08	0.00033	0.00207	6.00E-01	0.00452	0.00741	0.6	-0.0019	0.0085	8.20E-01	-0.0061	0.0065	3.46E-01	-0.0126	0.009	1.62E-01	0.0021	0.0082	7.94E-01	0.0036	0.002	8.89E-02	0.0048	0.0023	6.54E-02	0.0035	0.0015	1.13E-02
Short sleep	rs9321171	0.031	0.006	4.20E-08	-0.0028	0.00184	1.84E-03	0.00887	0.00659	0.19	-0.0036	0.0075	6.30E-01	-0.0014	0.0057	8.10E-01	-0.0058	0.0079	4.61E-01	0.0029	0.0073	6.92E-01	0.0027	0.0018	2.28E-01	-0.0011	0.0021	9.44E-01	-0.0001	0.0014	3.59E-01
Short sleep	rs9367621	0.024	0.006	1.60E-08	-0.009	0.00185	8.40E-06	-0.0055	0.00661	0.42	-0.0098	0.0075	1.90E-01	0.0049	0.0057	3.90E-01	0.0058	0.0079	4.62E-01	0.0044	0.0072	5.43E-01	0.0032	0.0018	1.18E-01	4.00E-04	0.0021	9.44E-01	-0.0023	0.0014	7.47E-02
Short sleep	rs11763750	0.035	0.008	5.10E-09	-0.0064	0.00235	7.50E-03	0.00364	0.00843	0.64	0.025	0.0093	6.30E-03	0.0277	0.007	8.10E-05	0.0226	0.01	2.32E-02	0.0207	0.0091	2.29E-02	0.0038	0.0022	1.58E-01	-3.00E-04	0.0025	5.25E-01	0.001	0.0016	6.50E-01
Short sleep	rs1229762	0.037	0.003	1.10E-12	0.0062	0.00193	3.20E-02	0.00083	0.00694	0.91	0.015	0.0079	6.50E-02	0.0239	0.0062	1.12E-04	0.0047	0.0083	5.74E-01	0.006	0.0079	4.49E-01	-7.00E-04	0.0019	8.36E-01	-0.0015	0.0022	5.61E-01	0.0018	0.0014	1.61E-01
Short sleep	rs60882754	0.055	0.012	1.80E-08	-0.0083	0.00386	4.50E-02	-0.0019	0.01364	0.85	-0.0092	0.016	5.70E-01	0.0284	0.012	1.83E-02	-0.0076	0.0178	6.68E-01	0.0132	0.016	4.09E-01	0.0031	0.0042	4.50E-01	-0.004	0.0048	5.15E-01	0.0038	0.003	2.36E-01
Short sleep	rs1607227	0.031	0.007	1.50E-09	-0.0027	0.00202	2.00E-01	-0.0018	0.00719	0.8	-8.00E-04	0.0082	9.20E-01	0.0041	0.0062	5.06E-01	0.0037	0.0086	6.71E-01	0.0094	0.0079	2.37E-01	-0.0017	0.0021	2.39E-01	0.0027	0.0023	5.12E-01	-0.0003	0.0015	8.64E-01
Short sleep	rs7939345	0.035	0.007	4.00E-08	-0.0122	0.00227	2.00E-06	0.01355	0.00807	0.089	0.019	0.0092	4.20E-02	-7.00E-04	0.0069	9.23E-01	-3.00E-04	0.01	9.80E-01	-0.0062	0.0091	4.93E-01	-0.0011	0.0022	5.98E-01	0.0029	0.0026	2.38E-01	-0.0007	0.0017	9.69E-01
Short sleep	rs17388803	0.053	0.01	6.50E-10	0.00569	0.00308	4.40E-02	-0.0101	0.01086	0.35	0.0037	0.012	7.60E-01	-0.0043	0.0093	6.48E-01	-0.0071	0.0125	5.71E-01	-0.005	0.0122	6.80E-01	0.0028	0.0031	5.22E-01	-0.005	0.0035	2.49E-01	9.00E-04	0.0022	2.16E-01
Short sleep	rs59779556	0.025	0.006	2.00E-08	0.0061	0.00186	5.00E-03	-0.0039	0.00659	0.58	0.0079	0.0075	2.90E-01	0.0043	0.0056	4.42E-01	-0.0015	0.0079	8.48E-01	-0.0044	0.0071	5.39E-01	3.00E-04	0.0018	7.42E-01	-0.0041	0.002	3.29E-02	0.0013	0.0013	3.48E-01
Short sleep	rs205024	0.031	0.006	2.70E-08	-0.0027	0.0019	3.20E-01	0.01586	0.00672	0.017	0.0095	0.0076	2.10E-01	5.00E-04	0.0058	9.31E-01	0.0113	0.008	1.59E-01	9.00E-04	0.0073	9.06E-01	-0.0016	0.0019	2.99E-01	0	0.0021	6.48E-01	-0.0029	0.0014	3.64E-02
Short sleep	rs12963463	0.029	0.006	1.90E-11	-0.0104	0.00205	7.70E-07	-0.0003	0.00723	0.95	-0.0087	0.0083	2.90E-01	0.0026	0.0062	6.76E-01	0.0113	0.0087	1.94E-01	0.0066	0.0078	3.94E-01	-0.0017	0.0019	1.43E-01	-0.0025	0.0021	2.17E-01	0.001	0.0014	4.49E-01
Short sleep	rs5757675	0.034	0.007	2.70E-09	0.00657	0.00213	2.50E-02	-0.0104	0.0075	0.15	0.022	0.0084	8.80E-03	0.0132	0.0065	4.32E-02	0.0013	0.0089	8.83E-01	0.0201	0.0083	1.55E-02	7.00E-04	0.002	7.55E-01	-0.0025	0.0023	3.47E-01	4.00E-04	0.0015	6.21E-01
Long sleep	rs7534398	0.047	0.012	2.10E-08	-0.0039	0.00229	5.00E-01	-0.0078	0.00819	0.37	0.0095	0.0093	3.10E-01	0.0017	0.0071	8.06E-01	-0.0148	0.01	1.41E-01	0.0071	0.009	4.27E-01	-6.00E-04	0.0023	6.77E-01	0.0045	0.0027	1.12E-01	9.00E-04	0.0017	3.43E-01
Long sleep	rs6737318	0.076	0.011	3.40E-13	-0.0076	0.00222	2.10E-03	0.00826	0.00793	0.22	0.0094	0.009	3.00E-01	-0.0013	0.0013	8.50E-01	-0.0093	0.0094	3.23E-01	0.0041	0.0088	6.40E-01	-0.0034	0.0022	5.98E-01	0.0026	0.0026	2.25E-01	-0.0033	0.0016	9.43E-02
Long sleep	#rs10899257	0.068	0.013	4.60E-08	-0.0048	0.00261	1.50E-01	-0.0093	0.00929	0.32	-0.025	0.011	1.90E-02	-0.0124	0.0079	1.16E-01	-0.0023	0.0112	8.37E-01	-0.0026	0.01	7.99E-01	0.0042	0.0026	2.16E-01	0.0011	0.003	7.90E-01	0.0006	0.0019	6.63E-01
Long sleep	rs3751046	0.07	0.013	2.00E-08	-0.0065	0.00261	4.10E-03	-0.004	0.00927	0.7	0.009	0.011	3.90E-01	-0.0037	0.0078	6.38E-01	0.012	0.0112	2.86E-01	0.0081	0.0098	4.05E-01	0.0047	0.0026	1.09E-01	-2.00E-04	0.003	7.56E-01	0.0017	0.0019	5.07E-01
Long sleep	rs75458655	0.185	0.029	5.40E-12	-0.0116	0.00616	4.50E-02	-0.0245	0.0219	0.28	-0.006	0.027	8.20E-01	-0.0096	0.0195	6.23E-01	-0.0121	0.0296	6.84E-01	0.0755	0.0284	7.86E-03	-0.0104	0.0074	7.81E-02	4.00E-04	0.0086	5.16E-01	-0.006	0.0055	1.62E-01
Long sleep	rs17817288	0.039	0.009	8.90E-09	-0.0172	0.00185	7.70E-17	0.01936	0.00654	0.0035	-0.043	0.0075	6.60E-09	-0.0165	0.0051	5.10E-03	-0.0348	0.0078	8.88E-06	-0.0137	0.0071	5.31E-02	0.0017	0.0016	5.97E-02	0.0017	0.0018	4.08E-01	-0.0056	0.0013	3.59E-05
Long sleep	rs17688916	0.071	0.012	1.10E-11	0.02347	0.00233	3.20E-19	-0.0365	0.00826	1.20E-05	-0.0056	0.0097	9.70E-03	-0.0232	0.0079	3.20E-03	-0.0133	0.011	2.24E-01	-0.0421	0.01	2.39E-05	0.005	0.0026	5.08E-02	0.0021	0.0032	3.11E-01	0.0035	0.0021	1.30E-01

*rs10068371 was used as a proxy for rs4585442 among all outcomes, while #rs11236879 was used as a proxy for rs10899257 among fasting glucose, fasting insulin and HbA1c. No proxy SNP was found for rs1380703 among eBMD.
